# Verrucomicrobial methanotrophs: ecophysiology of metabolically versatile acidophiles

**DOI:** 10.1093/femsre/fuab007

**Published:** 2021-02-01

**Authors:** Rob A Schmitz, Stijn H Peeters, Wouter Versantvoort, Nunzia Picone, Arjan Pol, Mike S M Jetten, Huub J M Op den Camp

**Affiliations:** Department of Microbiology, Institute for Water and Wetland Research, Radboud University, Heyendaalseweg 135, 6525 AJ, Nijmegen, The Netherlands; Department of Microbiology, Institute for Water and Wetland Research, Radboud University, Heyendaalseweg 135, 6525 AJ, Nijmegen, The Netherlands; Department of Microbiology, Institute for Water and Wetland Research, Radboud University, Heyendaalseweg 135, 6525 AJ, Nijmegen, The Netherlands; Department of Microbiology, Institute for Water and Wetland Research, Radboud University, Heyendaalseweg 135, 6525 AJ, Nijmegen, The Netherlands; Department of Microbiology, Institute for Water and Wetland Research, Radboud University, Heyendaalseweg 135, 6525 AJ, Nijmegen, The Netherlands; Department of Microbiology, Institute for Water and Wetland Research, Radboud University, Heyendaalseweg 135, 6525 AJ, Nijmegen, The Netherlands; Department of Microbiology, Institute for Water and Wetland Research, Radboud University, Heyendaalseweg 135, 6525 AJ, Nijmegen, The Netherlands

**Keywords:** verrucomicrobial methanotrophs, geothermal ecosystems, methane, proteobacterial methanotrophs, hydrogen gas, metabolism, comparative genomic analysis

## Abstract

Methanotrophs are an important group of microorganisms that counteract methane emissions to the atmosphere. Methane-oxidising bacteria of the Alpha- and Gammaproteobacteria have been studied for over a century, while methanotrophs of the phylum Verrucomicrobia are a more recent discovery. Verrucomicrobial methanotrophs are extremophiles that live in very acidic geothermal ecosystems. Currently, more than a dozen strains have been isolated, belonging to the genera *Methylacidiphilum* and *Methylacidimicrobium*. Initially, these methanotrophs were thought to be metabolically confined. However, genomic analyses and physiological and biochemical experiments over the past years revealed that verrucomicrobial methanotrophs, as well as proteobacterial methanotrophs, are much more metabolically versatile than previously assumed. Several inorganic gases and other molecules present in acidic geothermal ecosystems can be utilised, such as methane, hydrogen gas, carbon dioxide, ammonium, nitrogen gas and perhaps also hydrogen sulfide. Verrucomicrobial methanotrophs could therefore represent key players in multiple volcanic nutrient cycles and in the mitigation of greenhouse gas emissions from geothermal ecosystems. Here, we summarise the current knowledge on verrucomicrobial methanotrophs with respect to their metabolic versatility and discuss the factors that determine their diversity in their natural environment. In addition, key metabolic, morphological and ecological characteristics of verrucomicrobial and proteobacterial methanotrophs are reviewed.

## Teaser

This review discusses the metabolic versatility of verrucomicrobial methanotrophs regarding the acidic volcanic ecosystems they thrive in and a comparison is made with the canonical proteobacterial methanotrophs.

## INTRODUCTION

The atmospheric concentration of methane (CH_4_) has been increasing rapidly over the past quarter millennium due to anthropogenic activities (Etheridge *et al*. 1998; Turner, Frankenberg and Kort 2019). Currently, the amount of methane emitted from sources exceeds the amount of methane taken up by sinks, resulting in an imbalanced carbon cycle (Saunois *et al*. 2020). Consequently, the present atmospheric methane concentration (1.86 ppmv) is 2.6 times higher than the preindustrial concentration (Etheridge *et al*. 1998; Cai *et al*. 2016; Etminan *et al*. 2016; Saunois *et al*. 2020). Since methane is a powerful greenhouse gas, atmospheric methane is now estimated to contribute approximately 16% to global warming (Saunois *et al*. 2016). Moreover, an increase in global temperature can induce additional release of methane to the atmosphere, *e.g*. through permafrost thawing, causing positive climate feedback that results in an acceleration of climate change (Dean *et al*. 2018). Hence, it is important to understand the microbial sources and sinks of methane (Stein 2020).

Each year, 548 to 678 Tg (10^12^ g) CH_4_ is emitted from various sources into the atmosphere, of which 33%–54% and 46%–67% are from natural and anthropogenic origin, respectively (Kirschke *et al*. 2013; Dean *et al*. 2018). The majority of these sources contain methanogenic archaea that catalyse the final step of the biological degradation of organic matter by producing methane (Balch *et al*. 1979; Conrad 2020). The largest sources of methane are wetlands, which are anoxic, water-logged soils in which methanogenic archaea are present and active (Angel, Claus and Conrad 2012; Bridgham *et al*. 2013; Evans *et al*. 2019). Other natural sources of biogenic methane are aquatic systems (*e.g*. lakes), marine systems (especially coastal sediments), termites and wild animals (Jensen 1996; Reeburgh 2007; Bastviken *et al*. 2008; Brune 2010; Dean *et al*. 2018; Evans *et al*. 2019). Additional microbial processes that are implicated in methane production include conversion of methylphosphonates by Thaumarchaeota in the ocean and by cyanobacteria in freshwater and terrestrial ecosystems (Metcalf *et al*. 2012; Bižić *et al*. 2020). Natural sources in which methane is not produced microbially but through abiotic processes include wildfires and methane hydrates (located inside ocean floors and permafrost) (Buffett 2000; Vasileva and Moiseenko 2013; Dean *et al*. 2018). In addition, various geothermal systems such as fumaroles, mud volcanoes and hydrothermal vents emit 40 to 60 Tg CH_4_ annually (Etiope 2009). Methane released from these geothermal systems either has a thermogenic origin (*e.g*. formed through the thermogenic breakdown of organic matter over millions of years), an abiogenic origin (*e.g*. formed through the reduction of CO_2_ at high temperature in the Earth's crust) or even a biogenic origin, produced by methanogens in the deep subsurface (Sherwood Lollar, Lacrampe-Couloume and Slater 2006; Tassi *et al*. 2012; Stolper *et al*. 2015). As is the case for several natural sources, various man-made systems emit significant amounts of methane produced by methanogenic archaea. These sources include livestock (*e.g*. ruminants), landfills and rice paddies (Themelis and Ulloa 2007; Carlson *et al*. 2017; Dean *et al*. 2018; Chang *et al*. 2019). Other anthropogenic, non-biological sources are comprised of fossil fuels (through mining, combustion and industry) and biofuel/biomass (through combustion) (Hao and Ward 1993; Heede 2014; Hanaki and Portugal-Pereira 2018). The predominant sink conversion of methane occurs chemically in the troposphere by hydroxyl radicals (^•^OH) and to a lesser extent by chlorine and oxygen radicals (Allan, Struthers and Lowe 2007; Rigby *et al*. 2017). In various ecosystems, methane is produced in deeper anoxic layers that are covered by more oxidised sediments or water columns. In oxidised zones such as those present in wetlands, and notably at the oxic-anoxic interface, methanotrophic prokaryotes consume a major part of methane as energy and carbon source, prior to emission to the atmosphere (Brune, Frenzel and Cypionka 2000; Conrad 2009). These microorganisms therefore act as a biofilter for emissions of this potent greenhouse gas (La *et al*. 2018). In addition, specialised high-affinity methanotrophs present in soil seem to oxidise methane from the atmosphere, although only present at a very low concentration (Holmes *et al*. 1999; Cai *et al*. 2016; Tveit *et al*. 2019). Considering the role methanotrophs play in mitigating methane emissions, they are an important topic of study in understanding and counteracting global warming.

## METHANOTROPHIC MICROORGANISMS

Aerobic methanotrophs conserve energy by oxidising CH_4_ with O_2_ to CO_2_. The first aerobic methanotrophs were discovered and isolated more than a century ago (Kaserer 1905; Söhngen 1906). In the remainder of the twentieth century, numerous novel isolates were described, all belonging to the Alpha- and Gammaproteobacteria (Whittenbury, Phillips and Wilkinson 1970; Hanson and Hanson 1996). Methanotrophy is not restricted to oxic environments, but can take place anaerobically as well. Already decades ago, methane consumption in anoxic, sulfate-rich marine sediments was observed, but the microorganisms responsible for this process remained elusive (Barnes and Goldberg 1976; Reeburgh 1976; Iversen and Jorgensen 1985). At the beginning of the current century, a marine consortium of methane-oxidising archaea and sulfate-reducing bacteria was discovered, mediating sulfate-dependent methane oxidation (Boetius *et al*. 2000). This finding was followed by the discovery of a consortium of *Methylomirabilis* bacteria and *Methanoperedens* archaea, capable of coupling anaerobic methane oxidation to denitrification of nitrite and nitrate (Raghoebarsing *et al*. 2006). Hereafter it was shown that both the bacterium and the archaeon were capable of methane oxidation independently. *Methanoperedens* archaea are able to couple methane oxidation to the reduction of nitrate (Haroon *et al*. 2013), iron (Beal, House and Orphan 2009; Ettwig *et al*. 2016; Cai *et al*. 2018) and manganese (Beal, House and Orphan 2009; Ettwig *et al*. 2016; Leu *et al*. 2020), whereas *Methylomirabilis* bacteria (of the candidate phylum NC10) couple methane oxidation to the reduction of nitrite (Ettwig *et al*. 2008, 2010; He *et al*. 2016; Versantvoort *et al*. 2018). Remarkably, *Methylomirabilis* bacteria possess the complete aerobic methane oxidation pathway and are postulated to produce oxygen internally by reducing nitrite to nitric oxide, which is subsequently dismutated into O_2_ and N_2_ (Ettwig *et al*. 2010, 2012). Most aerobic and anaerobic methanotrophs live in environments with moderate temperature and circumneutral pH (Bowman *et al*. 1993). Exceptions are several aerobic proteobacterial methanotrophs of the genera *Methylocapsa, Methylocella, Methylocystis* and *Methylosinus* (Dedysh *et al*. 1998; Dunfield and Dedysh 2010; Kip *et al*. 2011). These bacteria are moderate acidophiles growing in acidic peat environments with a pH as low as 4.2 and are frequently found as intracellular symbionts of *Sphagnum* mosses (Raghoebarsing *et al*. 2005; Kostka, Weston and Glass 2016; Kox *et al*. 2018). In addition, methanotrophs of the gammaproteobacterial genus *Methylothermus* are thermophiles, growing in hot springs at temperatures up to 72°C (Bodrossy *et al*. 1999; Hirayama *et al*. 2011; Houghton *et al*. 2019). In 2005, methane oxidation was observed in volcanic soils of the Solfatara volcano (Campi Flegrei, Pozzuoli, Italy) that are characterized by a high temperature (50 to 95°C) and an extreme acidity (pH 1.0) (Castaldi and Tedesco 2005). Two years later, thermoacidophilic methanotrophs were isolated from hot and acidic volcanic ecosystems at the Solfatara volcano (near Naples, Italy), in the Uzon Caldera (Kamchatka, Russia) and in Hell's gate (Tikitere, New Zealand) (Dunfield *et al*. 2007; Pol *et al*. 2007; Islam *et al*. 2008). Surprisingly, phylogenetic analyses revealed that these microbes belong to the phylum Verrucomicrobia, refuting the dogma that all aerobic methanotrophs are part of the phylum Proteobacteria.

## METHANOTROPHS OF THE PHYLUM VERRUCOMICROBIA

**Figure 1.**
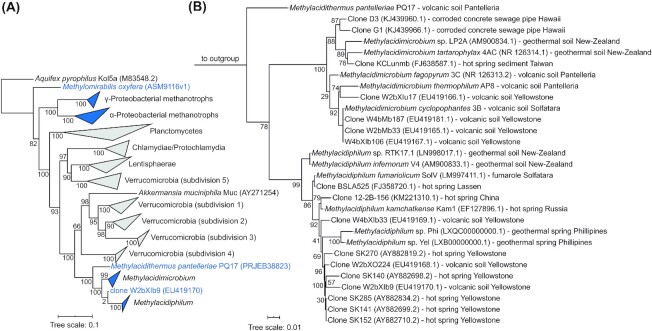
**(A)** Overview 16S rRNA gene phylogenetic tree of Verrucomicrobia, Lentisphaerae, Chlamydiae, Planctomycetes and methanotrophic Alpha- and Gammaproteobacteria. Blue shading indicates species that are methanotrophic. Accession numbers are indicated between brackets. 16S rRNA gene alignment was constructed using SINA v1.2.11 (Pruesse, Peplies and Glöckner 2012) and a tree that was constructed using FastTree v2.1 (Price, Dehal and Arkin 2010) with 1000 bootstraps and substitution model GTR-gamma. The tree was visualised using ITOL v5.5.1 (Letunic and Bork 2007). Bootstrap values are indicated as a proportion of 1000 re-samplings ranging from 1 to 100. **(B)** Detailed 16S rRNA gene tree of the verrucomicrobial methanotrophs. 16S rRNA gene alignment of verrucomicrobial methanotrophs together with an outgroup of Planctomycetes constructed using SINA v1.2.11 (Pruesse, Peplies and Glöckner 2012) and a tree that was constructed using FastTree v2.1 (Price, Dehal and Arkin 2010) with 1000 bootstraps and substitution model GTR-gamma. The tree was visualised using ITOL v5.5.1 (Letunic and Bork 2007). Bootstrap values are indicated as a proportion of 1000 re-samplings ranging from 1 to 100. Isolation location and accession number are indicated after each strain.

The first isolated verrucomicrobial methanotrophs were classified in the novel genus *Methylacidiphilum* (Op den Camp *et al*. 2009). These methanotrophs were observed to have temperature optima of 50 to 60°C (Dunfield *et al*. 2007; Pol *et al*. 2007; Islam *et al*. 2008). Currently, five *Methylacidiphilum* strains have been isolated that differ enough, based on DNA-DNA hybridisation and on the analysis of 16S rRNA and housekeeping genes, to be classified as separate species (Erikstad *et al*. 2019): *Methylacidiphilum fumariolicum* SolV, *Methylacidiphilum infernorum* V4, *Methylacidiphilum kamchatkense* Kam1, *Methylacidiphilum* sp. Yel and *Methylacidiphilum* sp. Phi (Table 1). The geothermal ecosystems they were isolated from have an extremely low pH, primarily as a result of the biogenic formation of sulfuric acid (H_2_SO_4_) from the oxidation of hydrogen sulfide (H_2_S) (Schoen and Rye 1970; Quatrini and Johnson 2018). Accordingly, the pH optima of all isolated *Methylacidiphilum* strains range from pH 2.0 to 3.0. Still, the pH range in which these strains can grow is much broader (Table 1). *Methylacidiphilum fumariolicum* SolV was shown to grow up to pH 6 when it was slowly adapted to higher pH (Mohammadi *et al*. 2017b). Moreover, this strain was shown to grow even below pH 1 (Pol *et al*. 2007) (Table 1). Through current culture-independent molecular methods, clones related to the isolated strains were found at geothermal sites across the globe, of which several could be novel species (Fig. 1).

**Table 1.** Summary of the pH and temperature optima, isolation location and verified growth substrates of the isolated verrucomicrobial methanotrophs discussed in this review.

Strain	pH_optimum_ (and range)	T_optimum_ (°C) (and range)	Verified growth substrates	Isolation location	References
*Methylacidiphilum fumariolicum* SolV	2 (0.8–6.0)	55 (40–65)	CH_4_, H_2_, methanol, propane, ethane	Acidic thermal mudpot, Solfatara, Italy	Pol *et al*. 2007; Mohammadi *et al*. 2017a; Picone *et al*. 2020b
*Methylacidiphilum infernorum* V4	2–2.5 (1.0–6.0)	60 (40–60)	CH_4_, methanol	Acidic thermal soil, Tikitere, New Zealand	Dunfield *et al*. 2007; Hou *et al*. 2008
*Methylacidiphilum kamchatkense* Kam1	2–2.5 (2.0–5.0)	55 (37–60)	CH_4_, methanol	Acidic thermal spring, Kamchatka, Russia	Islam *et al*. 2008
*Methylacidiphilum* sp. Phi	3	55–65	CH_4_	Acidic hot spring, Makiling, The Philippines	Erikstad *et al*. 2019
*Methylacidiphilum* sp. Yel	2.8	50	CH_4_	Acidic hot spring, Yellowstone, USA	Erikstad *et al*. 2019
*Methylacidimicrobium tartarophylax* 4AC	1–3 (0.5–5.5)	38 (?–43)	CH_4_, H_2_, methanol	Acidic soil, Solfatara, Italy	Van Teeseling *et al*. 2014; Mohammadi *et al*. 2019
*Methylacidimicrobium cyclopophantes* 3B	1.5–3 (0.6–5.5)	44 (?–49)	CH_4_, methanol	Acidic soil, Solfatara, Italy	Van Teeseling *et al*. 2014
*Methylacidimicrobium fagopyrum* 3C	1.5–3 (0.6–5.5)	35 (?–39)	CH_4_, methanol	Acidic soil, Solfatara, Italy	Van Teeseling *et al*. 2014
*Methylacidimicrobium* sp. LP2A	3.1 (1.0–5.2)	30 (17–37)	CH_4_	Acidic mud pool, Reporoa, New Zealand	Sharp *et al*. 2014
*Methylacidimicrobium thermophilum* AP8	3–5 (1.5–5.5)	50 (30–55)	CH_4_, H_2_	Acidic geothermal soil, Pantelleria island, Italy	Picone 2020

Since all isolated *Methylacidiphilum* strains were found in hot and acidic geothermal habitats, the question arose whether verrucomicrobial methanotrophs could have a more widespread distribution in habitats with different physicochemical parameters (Dunfield *et al*. 2007). Therefore, Sharp *et al*. (2014) conducted pyrosequencing of 16S rRNA genes on samples derived from geothermal habitats, acidic peat bogs and fens with a temperature range of 6.3 to 81.6°C and a pH range of 1.8 to 8.6. Surprisingly, 16S rRNA gene sequences of verrucomicrobial methanotrophs were detected in the full range of 22.5 to 81.6°C, but only below pH 5.0 (Sharp *et al*. 2014). From a sediment sample of 22°C and pH 2.6, the novel verrucomicrobial strain LP2A was enriched and isolated when incubated with methane as the sole energy source (Sharp *et al*. 2014). Interestingly, the 16S rRNA sequence of this newly isolated strain was only 90.6% identical to that of *Methylacidiphilum infernorum* V4, isolated from hot and acidic soil sediment (Dunfield *et al*. 2007). The novel mesophilic strain LP2A was subsequently placed in the novel genus *Methylacidimicrobium* within the phylum Verrucomicrobia (Van Teeseling *et al*. 2014). That same year, three additional mesophilic strains within this genus were isolated from geothermal soil at the Solfatara in Italy: *Methylacidimicrobium tartarophylax* 4AC, *Methylacidimicrobium fagopyrum* 3C and *Methylacidimicrobium cyclopophantes* 3B (Van Teeseling *et al*. 2014) (Fig. 1). These acidophiles have pH growth optima of 1.0 to 3.0, while strain 4AC can even grow at pH 0.5 (Van Teeseling *et al*. 2014) (Table 1). The major difference between *Methylacidiphilum* and *Methylacidimicrobium* strains was thought to be the growth temperature. *Methylacidimicrobium* strains were reported to be mesophiles, with temperature growth optima of 30 to 44°C and the apparent inability to grow at temperatures above 49°C (Sharp *et al*. 2014; Van Teeseling *et al*. 2014). However, recently, *Methylacidimicrobium thermophilum* AP8 was isolated from Pantelleria island (Italy) with a temperature optimum of 50°C (Picone 2020), questioning the clear temperature-based division between *Methylacidiphilum* and *Methylacidimicrobium* strains.

Apart from the strains isolated from geothermal soils in Italy, Russia and New-Zealand, 16S rRNA gene sequences belonging to strains of the *Methylacidimicrobium* genus were detected in hot springs and geothermal soil in other parts of the world (Fig. 1). In addition, a metagenome-assembled genome (MAG) representing a novel verrucomicrobial methanotroph genus and species, *Ca*. ‘Methylacidithermus pantelleriae’ PQ17, was obtained through sampling of geothermal soil on Pantelleria island (Picone *et al*. 2020a). Interestingly, verrucomicrobial methanotrophs do not seem to be restricted to geothermal habitats (Pagaling, Yang and Yan 2014). Clones related to *Methylacidimicrobium* strains were abundantly present in biofilms in the crown of corroded concrete sewage pipes on Hawaii, which suggests that verrucomicrobial methanotrophs have adapted to man-made systems (Pagaling, Yang and Yan 2014) (Fig. 1). These sewage pipes are characterised by a low pH and the presence of methane and sulfur compounds (*e.g*. H_2_S), which could explain the abundance of verrucomicrobial methanotrophs in this environment. Sequencing of samples from other acidic, methane-rich environments could reveal whether verrucomicrobial methanotrophs are present in other non-geothermal habitats.

Besides the importance of verrucomicrobial methanotrophs in the environment, they could also be relevant in biotechnology and industry, as they are capable of producing valuable molecules such as methanol, glycogen, polyhydroxybutyric acid, vitamins, compatible solutes and (thermostable) enzymes (Kalyuzhnaya, Puri and Lidstrom 2015; Strong, Xie and Clarke 2015; Bodelier *et al*. 2019). Although still in its infancy, notable examples of the biotechnological potential of verrucomicrobial methanotrophs are the recently shown improved methanol production (63% mole methanol produced per mole methane) by *Methylacidiphilum fumariolicum* SolV (Hogendoorn 2020; Hogendoorn *et al*. 2020) and the purification of a thermostable, high-affinity [NiFe] hydrogenase from the same bacterium (Schmitz *et al*. 2020a).

## KEY SIMILARITIES AND DIFFERENCES BETWEEN VERRUCOMICROBIAL AND PROTEOBACTERIAL METHANOTROPHS

Proteobacterial methanotrophs can be found in diverse oxic environments in which methane is present, such as wetlands, soil, peat lands, marine sediments, landfills and rice paddies (Bodelier *et al*. 2019). On the contrary, verrucomicrobial methanotrophs are almost exclusively found in acidic (pH < 3.5) geothermal systems. Whether proteobacterial methanotrophs are present in such acidic habitats is unknown. However, phylogenetic analyses of *pmoA* genes present in acidic geothermal soil and mud pots show clustering with proteobacterial *pmoA* gene sequences (Pol *et al*. 2007; Gagliano *et al*. 2014). This finding suggests that verrucomicrobial and proteobacterial methanotrophs could share habitats, which could ultimately be proven by the isolation of a proteobacterial methanotroph from an acidic geothermal ecosystem where *Methylacidiphilum* or *Methylacidimicrobium* were isolated from. Although mostly living in vastly different habitats, they share their preference for the greenhouse gas methane. Besides this metabolic trait, several noteworthy differences exist between the two groups. Classically, an important property for the classification of proteobacterial methanotrophs is the type of intracytoplasmic membrane (ICM) structures they have in electron micrographs, either visible as membrane pairs or vesicular discs (Davies and Whittenbury 1970). In verrucomicrobial methanotrophs, ICM structures are mostly absent, although membrane stacks have been found in *Methylacidimicrobium fagopyrum* 3C and also in some cells of *Methylacidiphilum infernorum* V4 tubular membrane structures were observed (Dunfield *et al*. 2007; Van Teeseling *et al*. 2014). Another interesting difference between verrucomicrobial and proteobacterial methanotrophs are the phospholipid fatty acids (PLFAs) of their membranes (Bodelier *et al*. 2009; Op den Camp *et al*. 2009). Membranes of verrucomicrobial methanotrophs are almost exclusively made up of saturated fatty acids, whereas membranes of proteobacterial methanotrophs are mainly composed of unsaturated fatty acids (Bodelier *et al*. 2009; Op den Camp *et al*. 2009; Erikstad *et al*. 2019). Verrucomicrobial methanotrophs probably require a saturated membrane to minimise proton permeability in an extremely acidic environment (Siliakus, van der Oost and Kengen 2017).

**Figure 2.**
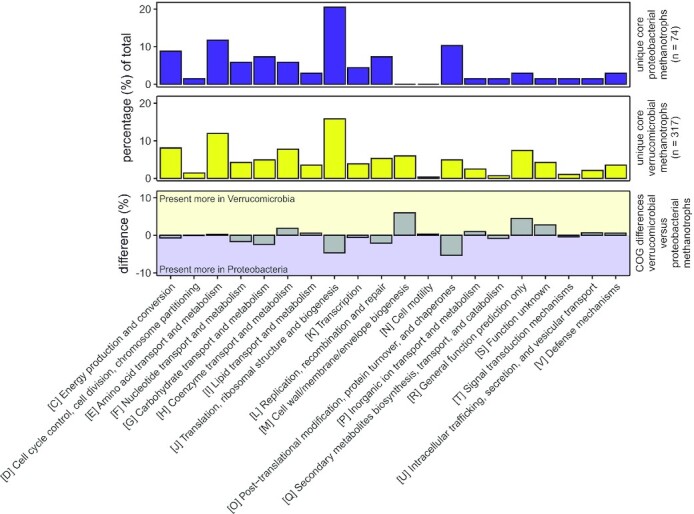
Bar graphs comparing the composition of Clusters of Orthologous Groups of proteins (COGS) of the unique core genomes of the verrucomicrobial methanotrophs and the proteobacterial methanotrophs. Core genomes were calculated by using usearch (Edgar 2010) with a cut-off value of 0.5 to group all genes into gene clusters. Clusters present in all genomes of the verrucomicrobial methanotrophs, as well as absent in all genomes of the proteobacterial methanotrophs, are defined as the unique core genome of verrucomicrobial methanotrophs. Clusters present in all genomes of the proteobacterial methanotrophs, as well as absent in all genomes of the verrucomicrobial methanotrophs, are defined as the unique core genome of proteobacterial methanotrophs. Analysis performed with 22 genomes in total, of which 11 verrucomicrobial methanotrophs: *Methylacidiphilum fumariolicum* SolV, *Methylacidiphilum infernorum* V4, *Methylacidiphilum kamchatkense* Kam1, *Methylacidiphilum* sp. Phi, *Methylacidiphilum* sp. Yel, *Methylacidimicrobium cyclopophantes* 3B, *Methylacidimicrobium fagopyrum* 3C, *Methylacidimicrobium tartarophylax* 4AC, *Methylacidimicrobium thermophilum* AP8, *Methylacidimicrobium* sp. LP2A and *Methylacidithermus pantelleriae* PQ17, and 11 proteobacterial methanotrophs: *Methylobacterium extorquens* AM1, *Methylocaldum szegediense* O-12, *Methylocapsa acidiphila* B2, *Methylocella silvestris* BL2, *Methylococcus capsulatus* Bath, *Methylocystis rosea* SV97T, *Methyloferula stellata* AR4, *Methylomarinum vadi* IT4, *Methylomicrobium alcaliphilum* 20Z, *Methylomonas denitrificans* FJG1 and *Methylosinus trichosporium* OB3b.

Remarkably, the genome sizes of verrucomicrobial and proteobacterial methanotrophs differ significantly. The former typically have a genome size of 2.2 to 2.5 Mbp (Hou *et al*. 2008; Anvar *et al*. 2014; Kruse *et al*. 2019; Cremers *et al*. 2020) while the latter have larger genomes of 3.3 to 5.1 Mbp (Ward *et al*. 2004; Chen *et al*. 2010; Stein *et al*. 2010; Boden *et al*. 2011; Svenning *et al*. 2011; Vuilleumier *et al*. 2012). The larger genomes of proteobacterial methanotrophs could render them better adapted to more diverse environments in comparison with verrucomicrobial methanotrophs (Cobo-Simón and Tamames 2017). Interestingly, genome comparison of eleven verrucomicrobial methanotrophs reveals 317 core gene clusters that are shared by all these verrucomicrobial strains, whereas the same analysis of eleven proteobacterial methanotrophs reveals 74 core gene clusters shared by all these proteobacterial strains (Fig. 2). The smaller amount of core gene clusters shared by proteobacterial methanotrophs could be explained by the fact that these microbes are found in very distinct habitats under various circumstances. The verrucomicrobial methanotrophs, on the other hand, are adapted to a specific niche. The composition of Clusters of Orthologous Groups of proteins (COGS) of the core genomes of verrucomicrobial and proteobacterial methanotrophs show a similar distribution (Fig. 2). A notable example is the relatively large percentage unique gene clusters involved in cell wall/membrane/envelope biogenesis (COG group M) found in verrucomicrobial methanotrophs. This might be explained by the harsh environment in which these microbes thrive, which could require additional machinery to cope with acid stress.

Although verrucomicrobial methanotrophs have smaller genomes, genomic analyses suggest verrucomicrobial methanotrophs are metabolically quite versatile microorganisms. In this review, we discuss the physiological and biochemical knowledge that has been obtained on the key metabolic pathways of verrucomicrobial methanotrophs since their discovery 13 years ago. As such, a connection between the metabolic potential in the genome and experimental observations is made. Metabolic genes investigated in this review involved in the oxidation of methane, ammonia, hydrogen and sulfur compounds, nitrogen assimilation, the respiratory chain and the synthesis of tetrahydrofolate and menaquinone can be found in Table S1 (Supporting Information).

## METABOLIC VERSATILITY OF VERRUCOMICROBIAL METHANOTROPHS

Initially, the first isolated strains appeared to be obligate methylotrophs, growing only on methane and methanol (CH_3_OH) (Op den Camp *et al*. 2009). In fact, all described *Methylacidiphilum* and *Methylacidimicrobium* strains were isolated on methane (Dunfield *et al*. 2007; Pol *et al*. 2007; Islam *et al*. 2008; Sharp *et al*. 2014; Erikstad *et al*. 2019). However, after more than a decade of additional experimental research, we now know that verrucomicrobial methanotrophs are actually metabolically quite versatile microorganisms, able to metabolise a variety of compounds present in volcanic ecosystems, which together could influence methanotrophy.

### Carbon and nitrogen assimilation

Similar to any other microorganism, methanotrophs need to assimilate carbon and nitrogen to generate biomass (Levicán *et al*. 2008). Methanotrophs use either formaldehyde (CH_2_O) or CO_2_ as a carbon source to generate biomass (Hanson and Hanson 1996). In general, gammaproteobacterial methanotrophs assimilate formaldehyde via the ribulose monophosphate pathway, whereas alphaproteobacterial methanotrophs employ the serine pathway, in which both formaldehyde and CO_2_ are used as a carbon source (Chistoserdova, Kalyuzhnaya and Lidstrom 2009). Verrucomicrobial methanotrophs lack a few essential genes for the complete serine cycle (*i.e*. hydroxypyruvate reductase and malyl-CoA lyase) or ribulose monophosphate pathway (*i.e*. 3-hexulose-6-phosphate synthase and 6-phospho-3-hexuloisomerase), but possess genes encoding the complete set of enzymes of the Calvin-Benson-Bassham (CBB) cycle (Khadem *et al*. 2011; Van Teeseling *et al*. 2014). In this cycle, the enzyme ribulose-1,5-bisphosphate carboxylase/oxygenase (RuBisCO) is involved and CO_2_ is used as the sole carbon source. Several proteobacterial methanotrophs also harbour genes involved in the CBB cycle, although activity of this cycle has not yet been experimentally validated in these bacteria (Stein, Roy and Dunfield 2012; Khmelenina *et al*. 2018). Through ^13^C stable isotope measurements, *Methylacidiphilum fumariolicum* SolV and three *Methylacidimicrobium* strains were shown to indeed exclusively use CO_2_ as carbon source, not methane-derived formaldehyde (Khadem *et al*. 2011; Van Teeseling *et al*. 2014). The autotrophic CO_2_ fixation via the CBB cycle was also experimentally shown for the denitrifying methanotroph *Ca*. ‘Methylomirabilis oxyfera’ (Rasigraf *et al*. 2014). In geothermal habitats, high concentrations of ammonium can be present (Khadem *et al*. 2010; Holloway *et al*. 2011; Mohammadi *et al*. 2017b). Although methanotrophs cannot use ammonium as an energy source, this reduced form of fixed nitrogen is the preferred inorganic nitrogen source as it can be directly assimilated (Wang *et al*. 2016). Indeed, all known verrucomicrobial methanotrophs carry a gene encoding a highly-conserved ammonium transporter. Central molecules in nitrogen assimilation are glutamate and glutamine, from which other amino acids and purines and pyrimidines can be synthesised (Prusiner and Stadtman 1973; Zalkin and Smith 1998). Genes encoding glutamate dehydrogenase, glutamine synthetase and glutamate synthase are found in all isolated verrucomicrobial methanotrophs. The utilisation of these three enzymes for nitrogen assimilation has been validated experimentally in several proteobacterial methanotrophs (Murrell and Dalton 1983a; Lees, Owens and Murrell 1991).

Although ammonium is often present in the geothermal environment, exponentially growing microorganisms can rapidly deplete this source of nitrogen from their direct surroundings (Van Heeswijk, Westerhoff and Boogerd 2013). If insufficient ammonium is present in the environment, many microorganisms have the possibility to take up nitrate or nitrite from the environment and invest energy to produce ammonium (Moreno-Vivián *et al*. 1999). All isolated *Methylacidiphilum* strains possess a gene encoding a transporter that could transport nitrate and/or nitrite across the membrane. In addition, they harbour a gene encoding a cytoplasmic NAD(P)H-dependent nitrate reductase that reduces nitrate to nitrite and a cytoplasmic NAD(P)H-dependent nitrite reductase that catalyses the 6-electron reduction to ammonium. The assimilatory reduction of nitrate to ammonium is catalysed at the expense of four NAD(P)H molecules and is therefore only used when ammonium in the environment is scarce. In several *Methylacidimicrobium* strains, the pathway for nitrate assimilation is unclear, or could be absent.

All isolated verrucomicrobial methanotrophs possess the *nifHDK* operon, indicating their ability to fix N_2_ from the atmosphere. *Methylacidiphilum fumariolicum* SolV and *Methylacidiphilum kamchatkense* Kam1 were experimentally shown to use N_2_ as nitrogen source (Islam *et al*. 2008; Khadem *et al*. 2010). Diazotrophy has also been shown for several proteobacterial methanotrophs (Murrell and Dalton 1983b; Khmelenina *et al*. 2018). Although the ability to obtain nitrogen from the atmosphere provides a major advantage in an environment devoid of fixed nitrogen molecules, the fixation of nitrogen gas is very energy-demanding, requiring 16 ATP molecules per N_2_ molecule fixed (Dixon and Kahn 2004). As a result, the maximum specific growth rate of *Methylacidiphilum fumariolicum* SolV with N_2_ as nitrogen source is 2.8 times slower than when supplied with ammonium (Khadem *et al*. 2010). The absence of fixed nitrogen in the environment might therefore lead to a reduction in growth of verrucomicrobial methanotrophs and therefore to increased methane emissions.

### Oxidation of methane and other hydrocarbons

Significant amounts of methane are emitted from mud pools, hot springs and fumaroles in terrestrial geothermal systems (Etiope and Klusman 2002; Castaldi and Tedesco 2005; Kvenvolden and Rogers 2005). Typically, geothermal gas consists of 0.01 to 1% (v/v) methane, although much higher concentrations have been detected (Giggenbach 1995; Etiope and Klusman 2002; D'Alessandro *et al*. 2009; Dunfield and Dedysh 2010). Under suitable conditions, verrucomicrobial methanotrophs could represent a significant filter against emissions of methane to the atmosphere in these environments (Op den Camp *et al*. 2009; Venturi *et al*. 2019). Studying in which circumstances verrucomicrobial methanotrophs consume more methane than in others is worth investigating to elucidate which factors enhance or inhibit methane oxidation.

**Figure 3.**
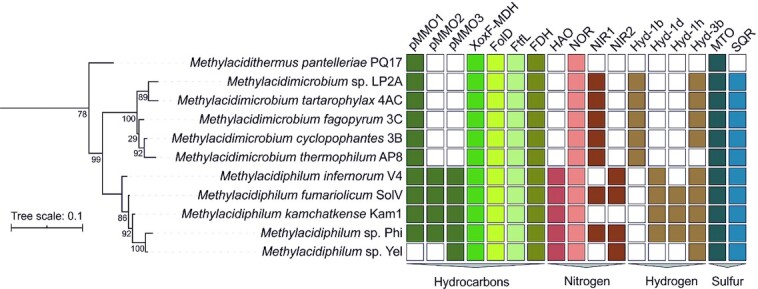
16S rRNA gene phylogenetic tree of verrucomicrobial methanotrophs and the presence or absence of genes involved in the oxidation of hydrocarbons, ammonia, hydrogen and sulfur compounds. Planctomycetes were used as an outgroup. Sequences were aligned using SINA v1.2.11 (Pruesse, Peplies and Glöckner 2012) and a tree was constructed using FastTree v2.1 (Price, Dehal and Arkin 2010) with 1000 bootstraps and substitution model GRT-GAMMA. The tree was visualised using ITOL v5.5.1 (Letunic and Bork 2007). Bootstrap values are indicated as a proportion of 1000 re-samplings ranging from 1 to 100. Presence of genes of interest was examined using BLASTp and of each hit the amino acid sequence was manually inspected for domains and identity. The result table was transformed to a binary ITOL dataset and both the 16S tree and the table were visualised using ITOL v5.5.1. pMMO: particulate methane monooxygenase; XoxF-MDH: lanthanide-dependent methanol dehydrogenase; FolD: methylenetetrahydrofolate dehydrogenase/methenyltetrahydrofolate cyclohydrolase; FtfL: formate-tetrahydrofolate ligase; FDH: formate dehydrogenase; HAO: hydroxylamine oxidoreductase; NOR: nitric oxide reductase; NIR: nitrite reductase; Hyd: type of [NiFe] hydrogenase (based on Søndergaard, Pedersen and Greening 2016); MTO: methanethiol oxidase; SQR: sulfide:quinone oxidoreductase.

**Figure 4.**
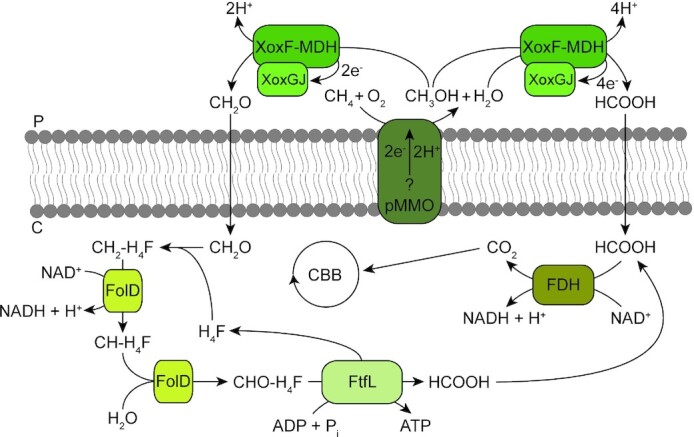
Possible pathways for methane oxidation in verrucomicrobial methanotrophs. pMMO oxidises methane to methanol (CH_3_OH), while an unknown electron donor is oxidised. The lanthanide-dependent XoxF methanol dehydrogenase (MDH) could subsequently oxidise methanol to either formate (HCOOH) or formaldehyde (CH_2_O), while donating electrons to its redox partner XoxGJ. If formate is produced it could diffuse into the cytoplasm and be converted to CO_2_ by the NAD^+^-dependent formate dehydrogenase (FDH). CO_2_ is fixed into biomass via the Calvin-Benson-Bassham (CBB) cycle. Alternatively, if formaldehyde is produced, it could bind to tetrahydrofolate (H_4_F) spontaneously or enzymatically by an unidentified enzyme, to form methylene-tetrahydrofolate (CH_2_-H_4_F). The enzyme FolD converts CH_2_-H_4_F to methenyl-tetrahydrofolate (CH-H_4_F), which is subsequently converted to formyl-tetrahydrofolate (CHO-H_4_F). This product is then converted to H_4_F and formate, while producing ATP. P: periplasm; C: cytoplasm.

The first step in methane oxidation is the conversion of methane to methanol (CH_3_OH), which can be catalysed by two genetically unrelated enzymes: the membrane-bound copper-containing particulate methane monooxygenase (pMMO) and the cytosolic iron-containing methane monooxygenase (sMMO) (Ross and Rosenzweig 2017). Several proteobacterial methanotrophs harbour genes encoding both the soluble and the particulate methane monooxygenase (Semrau, DiSpirito and Yoon 2010). However, none of the verrucomicrobial methanotrophs carry a gene encoding sMMO. *Methylacidiphilum* strains possess three complete but distinct *pmoCAB* operons and therefore in all probability oxidise methane to methanol via pMMO (Hou *et al*. 2008; Anvar *et al*. 2014; Van Teeseling *et al*. 2014; Kruse *et al*. 2019) (Figs 3 and 4). As exception, *Methylacidiphilum* sp. Yel only carries a single complete *pmoCAB* operon most closely related to the *pmoCAB3* of the other *Methylacidiphilum* strains. All pMMOs characterised thus far show an (αβγ)_3_ oligomeric state, consisting of the two membrane-spanning subunits PmoC and PmoA and a predominantly periplasmic subunit PmoB, which is tethered to the membrane by two transmembrane helices (Lieberman and Rosenzweig 2005; Ross and Rosenzweig 2017). The enzyme activates O_2_ to break the C-H bond of methane, producing methanol and water, via a yet unresolved mechanism (Ross and Rosenzweig 2017; Ross *et al*. 2019) (Fig. 4). Interestingly, similarities between two *pmo* operon structures of proteobacterial and verrucomicrobial methanotrophs indicate evolution from a common ancestor (Op den Camp *et al*. 2009; Van Teeseling *et al*. 2014). Besides, the most distant *pmoCAB3* operon seems to be obtained through horizontal gene transfer from an unknown microorganism (Dunfield *et al*. 2007). In fact, a large variety of key metabolic genes of verrucomicrobial methanotrophs are thought to be derived via horizontal gene transfer, especially from Proteobacteria (Sharp *et al*. 2013).

In *Methylacidiphilum fumariolicum* SolV, the oxygen concentration seems to regulate the expression of the *pmoCAB1* and *pmoCAB2* operons (Khadem *et al*. 2012). Notably, the *pmoCAB3* operon is barely expressed in *Methylacidiphilum fumariolicum* SolV when grown on methane (Khadem *et al*. 2012). Recently, *Methylacidiphilum fumariolicum* SolV was shown to grow on ethane and propane but not on butane (Picone *et al*. 2020b). *pmoCAB3* could be specifically tuned towards the oxidation of alkanes, enabling *Methylacidiphilum fumariolicum* SolV to utilise gaseous hydrocarbons other than methane (Picone *et al*. 2020b). However, *Methylacidiphilum* sp. Yel only carries *pmoCAB3* and is able to grow on methane, suggesting the enzyme encoded by this operon is at least also involved in the oxidation of methane (Erikstad *et al*. 2019). In contrast to *Methylacidiphilum* strains, *Methylacidimicrobium* strains and *Ca*. ‘Methylacidithermus pantelleriae’ PQ17 possess a single *pmoCAB* operon, related to the closely related *pmoCAB1* and *pmoCAB2* types of *Methylacidiphilum* species. As exception, *Methylacidimicrobium thermophilum* AP8 and *Methylacidimicrobium* sp. LP2A possess two almost identical operons (Sharp *et al*. 2014).

For a long time, the well-studied calcium-dependent methanol dehydrogenase (MDH) MxaFI was thought to be the key enzyme for methanol oxidation in both methanotrophs and methylotrophs (Anthony 2004). This periplasmic enzyme contains a pyrroloquinoline quinone (PQQ) prosthetic group at the catalytic centre and converts methanol to formaldehyde (CH_2_O) (Anthony 2004; Keltjens *et al*. 2014). The alphaproteobacterial methylotroph *Methylobacterium extorquens* AM1 carries genes encoding MxaFI and an MDH homolog named XoxF (Nakagawa *et al*. 2012). A Δ*mxaF* mutant strain was unable to grow on methanol when supplied with calcium, but growth on methanol was restored when cells were supplied with the lanthanide lanthanum (La^3+^) in the medium. Similarly, *Methylacidiphilum fumariolicum* SolV is only able to grow on methane when lanthanide-containing mud pot water (from the environment where the strain was isolated from) is added to the growth medium, or when the mineral medium contains micromolar concentrations of one or more lanthanides (Pol *et al*. 2014). These lanthanides are essential for verrucomicrobial methanotrophs when using methanol as an energy source, because they do not possess genes encoding the canonical calcium-dependent MxaFI, but a gene encoding the novel lanthanide-dependent MDH (XoxF) (Pol *et al*. 2014) (Fig. 3). Remarkably, the biological relevance of these rare earth elements was previously deemed unthinkable (Lim and Franklin 2004). Genomic analyses surprisingly revealed *xoxF* to be widespread in methylotrophs and methanotrophs, and is classified into five different clades (Chistoserdova 2011; Keltjens *et al*. 2014; Pol *et al*. 2014; Wu *et al*. 2015; Versantvoort *et al*. 2018; Kato *et al*. 2020). In fact, XoxF-type MDHs are actually much more abundant in nature than the MxaFI-type (Keltjens *et al*. 2014, Chistoserdova and Kalyuzhnaya 2018). The MxaF protein (large subunit) seems to have descended from a XoxF prototype (Keltjens *et al*. 2014) and all methylotrophs that harbour genes encoding MxaFI also possess a gene encoding the XoxF-type MDH (Chistoserdova and Kalyuzhnaya 2018). Dissolved lanthanide concentrations in most environments are very low (nM range) and its limitation may have driven the evolutionary adaptation of the proteobacterial methylotrophs towards an MDH variant that dependents on calcium, present in excess. The discovery of lanthanide-dependent XoxF-type MDHs has led to a completely new field of research, including the differential regulation in bacteria containing both types of MDHs (Zheng *et al*. 2018; Daumann 2019; Good *et al*. 2019; Picone and Op den Camp 2019; Good *et al*. 2020; Yanpirat *et al*. 2020). As example, the alphaproteobacterium *Methylobacterium extorquens* AM1 carries genes encoding both MDH types and was shown to upregulate *xoxF* expression already at a lanthanide concentration of 2.5 nM and downregulate *mxaF* expression at a lanthanide concentration of 25 nM or above (Vu *et al*. 2016; Daumann 2019).

Electrons yielded from methanol oxidation by XoxF are first transferred to a dedicated cytochrome partner (Zheng *et al*. 2018; Versantvoort *et al*. 2019) homologous to cytochrome *c_L_*, the electron acceptor of MxaFI-type MDH (Anthony 1992) (Fig. 4). In *Methylacidiphilum* strains, this cytochrome is a fusion protein of the cytochrome *c* protein XoxG and the periplasmic substrate binding protein XoxJ, but they also harbour a separate *xoxJ* gene. In *Methylacidimicrobium* strains, XoxG and XoxJ are encoded by two separate genes. *Methylacidiphilum* strains possess only one XoxF enzyme, whereas *Methylacidimicrobium* strains carry genes encoding for a copy of both the XoxF1 and XoxF2 clade that are less than 50% identical, which suggests that they could be expressed differentially under different conditions (Chistoserdova 2011; Keltjens *et al*. 2014; Picone and Op den Camp 2019). In addition, *Methylacidimicrobium* sp. LP2A harbours two genes encoding XoxF1 that are 96% identical to each other, presumably as a result of a relatively recent gene duplication (Van Teeseling *et al*. 2014; Op den Camp *et al*. 2018).

Similar to MxaFI-type MDH, XoxF-type MDH also contains a PQQ cofactor, and its overall structure is conserved in comparison with the MxaFI-type MDH (Keltjens *et al*. 2014). From *Methylacidiphilum fumariolicum* SolV, cultivated on lanthanide-containing mud pot water, XoxF was crystallised as a homodimer with a cerium ion coordinating with the PQQ cofactor (PDB: 4MAE; Pol *et al*. 2014). In addition, XoxF-type MDHs with a europium or lanthanum ion in the active site were crystallised (Deng, Ro and Rosenzweig 2018; Jahn *et al*. 2018; Good *et al*. 2020). The lanthanide coordinating with the PQQ cofactor of XoxF seems to render this enzyme catalytically more efficient compared to MxaF, since lanthanides are stronger Lewis acids than calcium, facilitating the hydride transfer (Keltjens *et al*. 2014; Daumann 2019). MxaFI-type MDHs produce formaldehyde from methanol oxidation, but XoxF was shown to produce formate (HCOOH) *in vitro* (Pol *et al*. 2014). Moreover, XoxF from *Methylacidiphilum fumariolicum* SolV can oxidise formaldehyde with high affinity and MxaFI-type MDHs in general are also able to oxidise formaldehyde (Keltjens *et al*. 2014). However, recently the XoxF5 of the alphaproteobacterium *Methylobacterium extorquens* AM1 was shown to oxidise methanol to formaldehyde *in vivo*, not to formate (Good *et al*. 2019). Currently, it is unresolved how formaldehyde would be oxidised further to formate in verrucomicrobial methanotrophs (Chistoserdova 2011). Formaldehyde could be oxidised directly to formate by formaldehyde dehydrogenase (Chistoserdova, Kalyuzhnaya and Lidstrom 2009), but verrucomicrobial methanotrophs do not possess a gene encoding this enzyme. Alternatively, methylotrophs can make use of the tetrahydromethanopterin (H_4_MPT) or the tetrahydrofolate (H_4_F) pathway for formaldehyde oxidation, as found in proteobacterial methanotrophs (Vorholt 2002; Marx, van Dien and Lidstrom 2005; Chistoserdova 2011). H_4_F is synthesised by bacteria *de novo* (Bermingham and Derrick 2002). Indeed, genes encoding all enzymes involved in tetrahydrofolate biosynthesis are present in the genomes of the verrucomicrobial methanotrophs. Formaldehyde produced by periplasmic XoxF could diffuse into the cytoplasm and bind spontaneously to H_4_F to form methylene-H_4_F (Kallen and Jencks 1966). Whether this condensation is of sufficient high rate for bacterial growth *in vivo* is under debate (Crowther, Kosály and Lidstrom 2008; He *et al*. 2020). In the proteobacterium *Methylobacterium extorquens* AM1, the formaldehyde-activating enzyme actually catalyses this reaction at high rate (Vorholt *et al*. 2000). Whereas this enzyme is present in proteobacterial methanotrophs, it is absent in verrucomicrobial methanotrophs, suggesting another enzyme should be involved in the condensation of formaldehyde and H_4_F. All verrucomicrobial methanotrophs carry a gene encoding the bifunctional enzyme FolD (Fig. 3), which is known to convert methylene-H_4_F to methenyl-H_4_F, and produce NAD(P)H, similar to the methylene-H_4_F dehydrogenase (Pawelek and MacKenzie 1998; Hou *et al*. 2008; Chistoserdova 2011; Eadsforth, Maluf and Hunter 2012). Subsequently, FolD acts as a methenyl-H_4_F cyclohydrolase, converting methenyl-H_4_F to formyl-H_4_F (Fig. 4). Ultimately, the enzyme formate-tetrahydroformate ligase FtfL, found in all verrucomicrobial methanotrophs, could convert formyl-H_4_F to tetrahydrofolate and formate, while producing one ATP molecule (Marx, Laukel and Vorholt 2003). If this pathway oxidises formaldehyde instead of XoxF, fewer electrons are donated to the cytochrome *c* protein XoxGJ, which is postulated to donate its electrons to the terminal oxidase to fuel respiration (Versantvoort *et al*. 2019). Nevertheless, the production of formaldehyde instead of formate by XoxF seems logical since formaldehyde oxidation via the H_4_F pathway provides valuable reducing equivalents in the form of NAD(P)H and ATP. Finally, in the last step of aerobic methane oxidation in verrucomicrobial methanotrophs, formate is oxidised to CO_2_ (Pol *et al*. 2014). All verrucomicrobial methanotrophs possess one complete *fdsDABG* operon, encoding a cytosolic formate dehydrogenase that is thought to oxidise formate, while producing NADH needed for the generation of biomass (Op den Camp *et al*. 2018). CO_2_ can ultimately be assimilated via the CBB cycle. The key enzyme RuBisCO found in verrucomicrobial methanotrophs forms a novel cluster within the form I RuBisCO phylogenetic tree and is most closely related to form IC RuBisCOs found in various Proteobacteria (Khadem *et al*. 2011).

### Coping with toxic nitrogen compounds from ammonia oxidation

In the geothermal habitat where *Methylacidiphilum fumariolicum* SolV was isolated from, high concentrations (1–28 mM) of ammonium (NH_4_^+^) are present (Khadem *et al*. 2010; Mohammadi *et al*. 2017b). Methane and ammonia (NH_3_) are highly-reduced molecules with structural similarities. Nitrifying microorganisms make a living from the oxidation of ammonia, initiated via the oxidation of ammonia to hydroxylamine (NH_2_OH), catalysed by ammonia monooxygenase (AMO) (Klotz and Stein 2008). AMO is a membrane-bound copper-dependent enzyme homologous to pMMO of methanotrophs (Tavormina *et al*. 2011). Consequently, many nitrifiers can oxidise methane and many methanotrophs can oxidise ammonia (Bédard and Knowles 1989). Methanotrophs use ammonium as nitrogen source; however, ammonia in the periplasm can also fortuitously be oxidised by pMMO to hydroxylamine, which is a toxic compound (Nyerges and Stein 2009). The affinity of pMMO for ammonia decreases with increasing CH_4_ concentrations, which indicates competitive substrate inhibition (Nyerges, Han and Stein 2010. Hydroxylamine could impede various cellular processes by damaging proteins or DNA and strongly inhibit MDHs (Kaplan and Ciotti 1953; Duine and Frank 1980; Versantvoort *et al*. 2020). Therefore, verrucomicrobial and proteobacterial methanotrophs have developed mechanisms to balance the assimilation of ammonium and the detoxification of deleterious nitrogen compounds.

**Figure 5.**
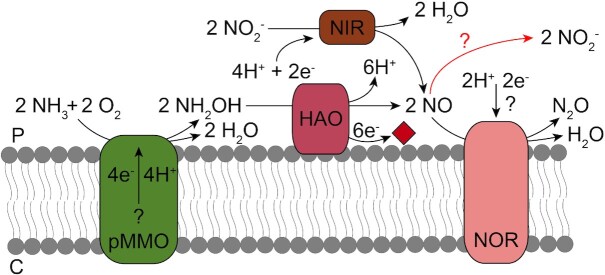
Ammonia (NH_3_) oxidation by pMMO and fate of the reaction products. pMMO fortuitously oxidises ammonia to hydroxylamine. Subsequently, hydroxylamine oxidoreductase (HAO) oxidises hydroxylamine to nitric oxide, donating electrons to an unknown cytochrome *c* protein (indicated by the red diamond). Nitric oxide can be reduced to inert N_2_O by the nitric oxide reductase (NOR), using an unknown electron donor. Alternatively, nitric oxide is converted chemically to nitrite in the presence of oxygen, or by an unknown enzyme (red arrow). Under anaerobic conditions, nitrite could be utilised as alternative electron acceptor. The nitrite reductase (NIR) could then reduce nitrite to nitric oxide, which is subsequently reduced to N_2_O. P: periplasm; C: cytoplasm.

In order to detoxify hydroxylamine, many methanotrophs possess a gene encoding a hydroxylamine oxidoreductase (HAO), oxidising hydroxylamine to nitric oxide (NO) (Fig. 5) (Caranto and Lancaster 2017; Versantvoort *et al*. 2020). All known *Methylacidiphilum* strains carry a gene encoding an HAO (Fig. 3), as do many proteobacterial methanotrophs (Campbell *et al*. 2011; Stein and Klotz 2011; Versantvoort *et al*. 2020). In contrast, HAO is not found in any of the *Methylacidimicrobium* strains and as such, these strains might be more sensitive to ammonium (Stein 2018). Alternatively, *Methylacidimicrobium* strains could have developed a different strategy to deal with ammonia that does not require an HAO, analogous to the gammaproteobacterial methanotroph *Methylomonas methanica*. This methanotroph also lacks an HAO-like protein (Campbell *et al*. 2011) and was unable to oxidise ammonia to nitrite, although its methane oxidation rate was inhibited by ammonia (Nyerges and Stein 2009). This could indicate that its pMMO is unable to oxidise ammonia to hydroxylamine or the formed hydroxylamine is converted to a product by an unknown enzyme other HAO. In either case, an HAO-like protein is not necessary to deal with ammonia toxicity. In nitrifiers, electrons yielded from the oxidation of hydroxylamine are transferred to the quinone pool for energy conservation via the hydroxylamine:ubiquinone reductase module (Klotz and Stein 2008). However, methanotrophs are thought to lack this module. Still, per ammonia molecule oxidised, four electrons are yielded when ammonia is oxidised to nitrite (Fig. 5). Since two electrons are needed to activate O_2_ for the oxidation of ammonia to hydroxylamine by pMMO, the remaining two electrons might be used at the terminal oxidase for energy conservation (Stein and Klotz 2011).

Nitric oxide produced by HAO is a toxic compound. However, when ammonia is oxidised by *Methylacidiphilum fumariolicum* SolV under aerobic conditions, nitrite is observed as the main end product (Mohammadi *et al*. 2017b), similar to aerobic ammonia oxidisers and various proteobacterial methanotrophs (Nyerges, Han and Stein 2010; Campbell *et al*. 2011; Lehtovirta-Morley 2018). These cases necessitate an additional enzyme that oxidises the NO produced by HAO to nitrite. The nature of this nitric oxide-oxidising enzyme in methanotrophs and ammonia-oxidisers is unknown, although it is postulated that the copper-dependent nitrite reductase NirK could function in the opposite direction to oxidise NO to nitrite (Caranto and Lancaster 2017). In addition, NO can rapidly react with oxygen resulting in the formation of nitrite and nitrate in aqueous solutions (Udert, Larsen and Gujer 2005; Hughes 2008) and therefore the production of nitrite observed for *Methylacidiphilum fumariolicum* SolV could also be non-enzymatic (Fig. 5). Under anoxic conditions, nitrite reduction rates in *Methylacidiphilum fumariolicum* SolV are higher compared with rates under oxic conditions (Mohammadi *et al*. 2017b). As such, the alternative electron acceptor nitrite could be converted to NO by a nitrite reductase, of which one or two orthologues are present in most verrucomicrobial methanotrophs (Fig. 3). Nitric oxide can subsequently be reduced to N_2_O, as was shown for the gammaproteobacterial methanotrophs *Methylomicrobium album* and *Methylomonas denitrificans* and for *Methylacidiphilum fumariolicum* SolV (Nyerges, Han and Stein 2010; Kits, Klotz and Stein 2015; Mohammadi *et al*. 2017b). The membrane-bound nitric oxide reductase NorCB reduces NO to N_2_O and is found in all known verrucomicrobial methanotrophs (Figs 3 and 5). It is therefore conceivable that verrucomicrobial methanotrophs detoxify nitric oxide to inert N_2_O under anoxic or oxygen-limited conditions (Acton and Baggs 2011; Bodelier and Steenbergh 2014). In addition, several proteobacterial methanotrophs were shown to produce N_2_O without the initial formation of nitrite (Hoefman, van der Ha and Boon 2014). Since N_2_O is strong greenhouse gas with a significant role in global warming, methanotrophs might not only have a mitigating effect on climate change by oxidising methane, but also aggravate this change by producing N_2_O (Acton and Baggs 2011; Versantvoort *et al*. 2020). Altogether, the accidental oxidation of ammonia by pMMO results in the formation of various toxic compounds, for which *Methylacidiphilum* strains typically appear to have multiple detoxification pathways.

### Oxidation of hydrogen gas

Multiple proteobacterial methanotrophs carry hydrogenase genes and were shown to consume H_2_ (De Bont 1976; Csáki *et al*. 2001; Kelly, Anthony and Murrell 2005). These methanotrophs were proposed to use these hydrogenases to produce reducing equivalents to drive the energy-demanding conversion of methane to methanol by pMMO (Hanczár *et al*. 2002). Besides, various proteobacterial methanotrophs possess genes encoding both a hydrogenase and ribulose-1,5-biphosphate carboxylase/oxygenase (RuBisCO), suggesting autotrophic growth on H_2_ is possible (Mohammadi *et al*. 2019). However, autotrophic growth on H_2_ in liquid media without methane has never been observed for proteobacterial methanotrophs (Taylor, Dalton and Dow 1981; Baxter *et al*. 2002).

Hydrogen gas (H_2_) is typically emitted in geothermal habitats in high concentrations (often > 1% v/v) and is a potential alternative energy source for various microorganisms (Aragno 1992; Chiodini *et al*. 2001; Carere *et al*. 2017; Mohammadi *et al*. 2019). Indeed, *Methylacidiphilum fumariolicum* SolV was experimentally shown to make a living as autotrophic hydrogenotroph in the absence of methane (Mohammadi *et al*. 2017a). Since methane emissions can heavily fluctuate in geothermal habitats, the ability to grow on another emitted, energy-rich gas is highly advantageous. *Methylacidiphilum* sp. RTK17.1 (an isolate almost identical to *Methylacidiphilum infernorum* V4) was shown to grow as a mixotroph on H_2_ and CH_4_, a trait hypothesised to have driven niche expansion, which could explain the dominance of verrucomicrobial methanotrophs in geothermal habitats (Carere *et al*. 2017; Mohammadi *et al*. 2019). Recently, this mixotrophic lifestyle on H_2_ and CH_4_ was also demonstrated by the alphaproteobacterium *Methylocystis* sp. strain SC2 (Hakobyan, Zhu and Glatter 2020).

Hydrogenases are a very diverse group of enzymes that convert H_2_ into two protons and two electrons, or vice versa (Lubitz *et al*. 2014). Three different metal compositions are known in the active site: [NiFe], [FeFe] and [Fe]. Genome comparisons of several verrucomicrobial methanotrophs revealed that all these microbes carry genes encoding one or more different [NiFe] hydrogenases, except for *Ca*. ‘Methylacidithermus pantelleriae’ PQ17 (Fig. 3) (Mohammadi *et al*. 2019). It has to be noted that the genome of strain PQ17 was retrieved from a metagenome, not from an isolate. It might therefore still possess several genes that are not directly found through genetic analyses, although the genome is more than 98% complete and shows little contamination (Picone 2020). All isolated *Methylacidiphilum* strains carry genes encoding the group 1d [NiFe] hydrogenase, except for *Methylacidiphilum* sp. Yel. This membrane-bound enzyme has a relatively high O_2_ tolerance compared to other hydrogenases and is involved in the aerobic respiration of H_2_ (Greening *et al*. 2015). Both *Methylacidiphilum* sp. RTK17.1 and *Methylacidiphilum fumariolicum* SolV were experimentally shown to grow on H_2_ using this hydrogenase under various oxygen concentrations (Carere *et al*. 2017; Mohammadi *et al*. 2017a).

All of the *Methylacidimicrobium* strains and none of the *Methylacidiphilum* strains possess genes encoding the oxygen-sensitive group 1b [NiFe] hydrogenase (Fig. 3) (Mohammadi *et al*. 2019). *Methylacidimicrobium tartarophylax* 4AC was shown to grow as an autotroph on H_2_ using this enzyme under microoxic conditions (Mohammadi *et al*. 2019). The group 1b [NiFe] hydrogenase, typically found in Proteobacteria that perform anaerobic respiration (Greening *et al*. 2015), therefore enables *Methylacidimicrobium* strains to respire H_2_ aerobically. In addition, all *Methylacidimicrobium* and *Methylacidiphilum* strains, except for *Methylacidimicrobium thermophilum* AP8, carry genes encoding a group 3b [NiFe] hydrogenase. This heterotetrameric cytosolic enzyme is known to couple the oxidation of NADPH to the production of H_2_ during fermentation (Berney *et al*. 2014). However, in the verrucomicrobial methanotrophs it is hypothesised to oxidise H_2_ and produce NADH for CO_2_ fixation (Carere *et al*. 2017). This proposed catalysis was demonstrated by the group 3b [NiFe] hydrogenase of *Hydrogenobacter thermophilus* (Yoon *et al*. 1996).

Most hydrogen-oxidising microorganisms live in habitats with relatively high H_2_ concentrations, such as animal guts and leguminous soils (Conrad and Seiler 1979; Pester and Brune 2007). In addition, several soil-inhabiting Actinobacteria were shown to consume H_2_ present in the atmosphere (Constant, Poissant and Villemur 2008; Constant *et al*. 2010; Constant *et al*. 2011). Although the atmospheric H_2_ concentration is very low (0.53 ppmv H_2_), soil systems are the largest sink of atmospheric H_2_, consuming 75 Tg H_2_ annually (Novelli *et al*. 1999). These H_2_ scavengers possess genes encoding a putative high-affinity [NiFe] hydrogenase, of which the oxygen-tolerant group 1h [NiFe] hydrogenase seems to be dominant. Remarkably, this group 1h [NiFe] hydrogenase is also found in the genomes of *Methylacidiphilum fumariolicum* SolV, *Methylacidiphilum kamchatkense* Kam1 and *Methylacidiphilum* sp. Phi (Fig. 3) (Mohammadi *et al*. 2017a; Kruse *et al*. 2019). In the related *Methylacidiphilum infernorum* V4, this gene may have been lost over time due to the movement of transposable elements in the genome (Kruse *et al*. 2019). *Methylacidiphilum fumariolicum* SolV was shown to express both the group 1d and group 1h [NiFe] hydrogenase to grow on H_2_ (Mohammadi *et al*. 2017a). At dissolved oxygen concentrations between 0.2 and 1.5%, only the group 1h [NiFe] hydrogenase supported growth, up to a growth rate of 0.03 h^−1^, which is almost 40% of the growth rate on methane (Mohammadi *et al*. 2017a). The enzyme has a remarkable tolerance towards O_2_, suggested to be the result of the unique coordination of the proximal [4Fe4S] clusters by three cysteines and an aspartate residue, instead of the usual coordination by four cysteine residues (Schäfer, Friedrich and Lenz 2013; Schäfer *et al*. 2016). The group 1h [NiFe] hydrogenase was purified from *Methylacidiphilum fumariolicum* SolV and kinetic experiments revealed an unusually high affinity for H_2_, enabling this methanotroph to oxidise atmospheric H_2_ (Schmitz *et al*. 2020a). It is hypothesised that this hydrogenase in particular could aid verrucomicrobial methanotrophs to thrive in geothermal systems (Carere *et al*. 2017; Schmitz *et al*. 2020a).

### Oxidation of sulfur compounds

A variety of inorganic sulfur compounds such as hydrogen sulfide (H_2_S) and sulfur dioxide (SO_2_) are present in or released from geothermal systems (Spiro, Jacob and Logan 1992; Vasilakos *et al*. 2005). Additionally, organic sulfur compounds such as methanethiol (CH_3_SH) could be present, but data on its presence in terrestrial volcanic ecosystems are lacking. In seafloor hydrothermal systems, methanethiol is formed abiotically from CO_2_, H_2_S and H_2_, derived from organic matter below the ocean floor (Rogers and Schulte 2012; Reeves *et al*. 2014). It is conceivable that in terrestrial mud volcanoes with comparable conditions, thermogenic production of methanethiol could occur as well. This compound could then be used by methylotrophs such as the verrucomicrobial methanotrophs as carbon and sulfur source, or even as energy source, as observed in the methylotrophic alphaproteobacterium *Hyphomicrobium* sp. VS (Pol *et al*. 1994). Studying (organic) sulfur compounds and how microorganisms cope with these compounds is important, as several of these compounds have profound effects on the environment, causing acid precipitation and cloud formation (Lomans, Pol and Op den Camp 2002).

Little is known about the utilisation of sulfur compounds as energy sources in methanotrophs. Genomic comparison of verrucomicrobial methanotrophs revealed that all known strains carry a gene encoding methanethiol oxidase (annotated as selenium-binding protein 56) (Eyice *et al*. 2018) (Fig. 3). This enzyme oxidises methanethiol to form formaldehyde, hydrogen sulfide and hydrogen peroxide (H_2_O_2_) (Suylen *et al*. 1987). The oxidation of methanethiol could render the verrucomicrobial methanotrophs with useful products. Formaldehyde could be converted to formate via FolD and FtfL and to CO_2_ by the formate dehydrogenase (Fig. 4). H_2_S can be used as a sulfur source and could even be used as an energy source. In addition, a gene encoding sulfide:quinone oxidoreductase (SQR) is found in the genomes of all verrucomicrobial methanotrophs, and highly expressed in *Methylacidiphilum fumariolicum* SolV (Mohammadi *et al*. 2017a). More specifically, it contains a type III SQR of which little is known, that could be involved in aerobic H_2_S respiration (Marcia, Ermler and Peng 2010). The third product of methanethiol oxidation, hydrogen peroxide, is toxic and could be converted to water and oxygen by the enzyme catalase (Zamocky, Furtmüller and Obinger 2008). A gene encoding catalase is only found in *Methylacidiphilum infernorum* V4 and all *Methylacidimicrobium* strains. However, several peroxidases have been found in the genomes that might substitute the catalase activity.

The effect of intracellular H_2_S and methanethiol in methanotrophs is currently not well understood. In some microorganisms, methane oxidation seems to be enhanced or unaffected by the presence of methanethiol or hydrogen sulfide (Lee, Kim and Cho 2012), whereas in other strains methane oxidation is inhibited by these compounds (Börjesson 2001; Lee *et al*. 2015). Since all verrucomicrobial methanotrophs possess a gene encoding an SQR and because H_2_S is generally emitted at high concentrations from geothermal systems (Chiodini *et al*. 2001), it is conceivable that also H_2_S could be an important energy source for these microorganisms. Alternatively, sulfide-oxidising enzymes could be present as a means of detoxification. Future experiments have to resolve whether energy conservation from the oxidation of H_2_S is indeed possible.

### The electron transport chain and energy conservation

The main location for energy conservation in prokaryotic cells is the cytoplasmic membrane. Quinones inside the cytoplasmic membrane are essential parts of the electron transport chain and therefore essential to energy conservation (Kurosu and Begari 2010). Verrucomicrobial methanotrophs seem to synthesise menaquinones (MK) via an alternative pathway that involves the intermediate futalosine (Hiratsuka *et al*. 2008). However, the gene *mqnB* involved in menaquinone biosynthesis was not detected in any of the genomes of verrucomicrobial methanotrophs (Kruse *et al*. 2019), but pathway variations are known to exist (Arakawa *et al*. 2010).

**Figure 6.**
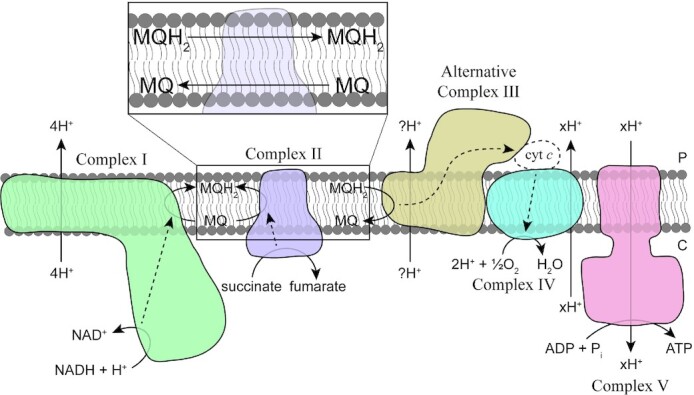
Schematic overview of the typical electron transport chain of verrucomicrobial methanotrophs. NADH generated through catabolic processes is oxidised by the NADH:quinone oxidoreductase (Complex I), transferring electrons to menaquinone (MQ) to form menaquinol (MQH_2_) while translocating protons across the membrane to generate a proton motive force. The succinate dehydrogenase (Complex II) oxidises succinate to fumarate as a part of the tricarboxylic acid cycle and transfers electrons to MQ to form MQH_2_. Alternative complex III oxidises MQH_2_ back to MQ, transferring electrons to a cytochrome *c* oxidase (Complex IV), either via an external periplasmic cytochrome *c* protein (cyt *c*) or directly. Whether protons are translocated during this electron transfer is unknown. Ultimately, electrons are used to reduce O_2_ to water, while protons are translocated over the membrane, contributing to the proton motive force that is used by an ATP synthase (Complex V) to synthesise ATP. Dashed lines indicate electron flow. Inset: transfer of oxidized and reduced quinones inside the bilayer (the quinone pool). C: cytoplasmic side of the membrane; P: periplasmic side of the membrane.

All verrucomicrobial methanotrophs described in this review possess five different complexes involved in energy conservation (Fig. 6). The NADH:quinone oxidoreductase (Complex I) is a membrane-bound protein complex that oxidises NADH, transfers the freed electrons to the quinone pool, and couples this reaction to the translocation of protons across the membrane, contributing to a proton motive force (Friedrich and Scheide 2000). The bacterial enzyme typically consists of 14 subunits, which are all encoded by the verrucomicrobial methanotrophs (Berrisford, Baradaran and Sazanov 2016). Complex II, or succinate dehydrogenase, is involved in both the electron transport chain and the tricarboxylic acid (TCA) cycle (Cecchini *et al*. 2002). In this cycle, Complex II couples the oxidation of succinate to the reduction of quinone (Fig. 6). Whereas Complex I is also involved in the translocation of protons across the membrane, Complex II is only involved in electron transfer. The quinols produced from the reduction of quinones by both complexes are typically oxidised by the *bc*_1_ complex (Complex III) (Trumpower 1990). In that case, electrons yielded from the oxidation of quinols are subsequently transferred via an additional cytochrome *c* protein to a cytochrome *c* oxidase (complex IV or terminal oxidase). However, the verrucomicrobial methanotrophs do not carry genes encoding Complex III. Instead, these microorganisms possess genes encoding a structurally different protein complex with similar function: the Alternative Complex III (ACIII; Fig. 6) (Pereira *et al*. 2007; Refojo *et al*. 2010). The classical Complex III contributes to the proton motive force by translocating protons over the membrane via the quinone cycle. Whether ACIII also translocates protons across the membrane for energy conservation is under debate (Refojo, Teixeira and Pereira 2012; Sun *et al*. 2018). Ultimately, Complex IV reduces the terminal electron acceptor O_2_ to water, with electrons retrieved from a *c*-type haem (Sun *et al*. 2018). All isolated verrucomicrobial methanotrophs, except for *Ca*. ‘Methylacidithermus pantelleriae’ PQ17, possess genes encoding three distinct Complexes IV: a *cbb*_3_-type, an *aa*_3_-type and a *ba*_3_-type. These cytochrome *c* oxidases are classified based on the type of haems they contain and are known to have different affinities for O_2_ and can be differentially used for various substrates (García-Horsman *et al*. 1994). In addition, *Methylacidiphilum fumariolicum* SolV and *Methylacidiphilum* sp. Phi possess a second *ba*_3_-type cytochrome *c* oxidase. Interestingly, ACIII of *Flavobacterium johnsoniae* can form a supercomplex with Complex IV, in which an additional cytochrome *c* protein for electron transfer is not needed (Sun *et al*. 2018). With this in mind, it is interesting to note that in verrucomicrobial methanotrophs the genes encoding the different subunits composing ACIII are found directly adjacent to the genes encoding the *cbb*_3_-type cytochrome *c* oxidase. Likewise, in other microorganisms the genes encoding ACIII are often found directly adjacent to genes encoding a cytochrome *c* oxidase (Refojo *et al*. 2010). However, only in verrucomicrobial methanotrophs and Opitutaceae a C-type cytochrome *c* oxidase is found together with ACIII, whereas typically these are A-type or B-type cytochrome *c* oxidases (Refojo *et al*. 2010). The different cytochrome *c* oxidases encoded by *Methylacidiphilum fumariolicum* SolV are differentially expressed, underscoring the metabolic versatility of verrucomicrobial methanotrophs (Khadem *et al*. 2012; Mohammadi *et al*. 2017a). Interestingly, the *ba*_3_-type cytochrome *c* oxidase of *Aquifex aeolicus* is able to use ubiquinol as electron donor, reduced by electrons yielded from sulfide oxidation by the sulfide:quinone oxidoreductase (SQR) (Gao *et al*. 2012). Since the verrucomicrobial methanotrophs encode for both a *ba*_3_-type cytochrome *c* oxidase and SQR, these bacteria might utilise a specific system to conserve energy from H_2_S oxidation, but this needs experimental validation. The proton motive force created by the transfer of electrons through the different membrane complexes is used for the synthesis of ATP via the ATP synthase (Complex V). The verrucomicrobial methanotrophs, except for *Ca*. ‘Methylacidithermus pantelleriae’ PQ17, carry genes encoding two ATP synthases (ATPases). One ATPase is related to ATPases of other Verrucomicrobia, while the other is related to the gammaproteobacterial ATPase (Hou *et al*. 2008; Kruse *et al*. 2019).

## VERRUCOMICROBIAL METHANOTROPHS IN THE ENVIRONMENT

Verrucomicrobial methanotrophs are generally found in acidic volcanic ecosystems. Interestingly, global biogeographic distributions of closely related strains of verrucomicrobial methanotrophs suggest allopatric evolution over time (Erikstad *et al*. 2019). Additionally, evolutionary genomic analyses of verrucomicrobial methanotrophs revealed multiple horizontal gene transfer events (Sharp *et al*. 2013). Many genes have a relatively high similarity to those of the phylum Proteobacteria, but also to genes of Aquificae, Thermus/Deinococcus and archaea (Sharp *et al*. 2013; Schmitz *et al*. 2020b). Over the past 13 years, physiological and biochemical experiments have rendered valuable information that allows us to predict in which habitats we could encounter verrucomicrobial methanotrophs. Ecophysiological experiments could shed light on how verrucomicrobial methanotrophs interact with other microorganisms in the natural environment.

Acidic volcanic ecosystems are characterised by various morphological features. In general, these features are formed in places where mud, gas and water are expelled due to high fluid pressure from the subsurface, creating hot springs, mud pools and fumaroles (Cioni, Corazza and Marini 1984; Feyzullayev and Movsumova 2010; Benson *et al*. 2011). Where reduced sulfur compounds are present, often sulfuric acid (H_2_SO_4_) is produced by (thermo)acidophilic microorganisms. The microbial production of sulfuric acid is environmentally important because it creates a low pH, which is characteristic for the Solfatara, several parts of Yellowstone National Park and various other geothermal ecosystems (Schoen and Rye 1970; Quatrini and Johnson 2018). A low pH seems to be a key determinant of verrucomicrobial methanotrophs to be present in geothermal habitats (Sharp *et al*. 2014). 16S rRNA and *pmoA* gene sequences of verrucomicrobial methanotrophs have so far not been detected in habitats with a pH > 5.0 and all isolates have a pH optimum around 3. Physicochemical parameters within geothermal habitats such as temperature and the oxygen concentration can differ locally within a few meters. Many of the thermophilic *Methylacidiphilum* strains were isolated from hot geothermal areas in close proximity to the moderate temperature geothermal areas where the *Methylacidimicrobium* strains were isolated from (Sharp *et al*. 2014). Although bogs and fens are also characterised by a high acidity (pH 3.3 to 4.9), no 16S rRNA or *pmoA* gene sequences of verrucomicrobial methanotrophs have been detected in these habitats, which may be explained by the relatively low concentration of lanthanides in peat bogs (Vodyanitskii *et al*. 2012; Sharp *et al*. 2014). Based on environmental sequencing and activity studies, the methanotrophic activity can be attributed to Proteobacteria, at a pH as low as 3.5 (Dunfield and Dedysh 2010). Proteobacteria such as strains of the genus *Acidithiobacillus* have been found at lower pH, but this does not include proteobacterial methanotrophs (Crognale *et al*. 2018). With current knowledge, methane-rich habitats below pH 3.5, including the aforementioned crown of rusted sewer pipes, seem to be better suited for verrucomicrobial methanotrophs than proteobacterial methanotrophs.

Methane and hydrogen gas are often emitted from geothermal habitats and these two compounds seem to be the major energy sources for verrucomicrobial methanotrophs. All verrucomicrobial methanotrophs were isolated on methane and several strains were shown to grow as autotrophs on H_2_ (Carere *et al*. 2017; Mohammadi *et al*. 2017a; Mohammadi *et al*. 2019). Multiple verrucomicrobial methanotrophs possess more than one particulate methane monooxygenase. The three *pmo* operons within the *Methylacidiphilum* strains differ significantly in amino acid sequences, suggesting selection pressure for different functions and adaptation to changing environmental conditions (Op den Camp *et al*. 2009). In addition, short chain alkenes could be oxidised by one or more of the pMMOs found in *Methylacidiphilum* strains (Picone *et al*. 2020b). For methane oxidation to occur, a sufficient concentration of lanthanides must be present in the environment, because the verrucomicrobial methanotrophs only carry genes encoding the lanthanide-dependent XoxF-type methanol dehydrogenase. This does not necessarily mean that verrucomicrobial methanotrophs are only present in lanthanide-rich environments. If H_2_ and oxygen are present at suitable concentrations, they could grow as Knallgas bacteria without the need for lanthanides (Carere *et al*. 2017; Mohammadi *et al*. 2017a; Mohammadi *et al*. 2019). The concentration of H_2_ and O_2_ in the environment could partially determine which strains of verrucomicrobial methanotrophs are present in the environment, because the different kinds of [NiFe] hydrogenases found in their genomes may have very different tolerances towards oxygen and possess different affinities for H_2_. As example, *Methylacidimicrobium tartarophylax* 4AC only respires H_2_ at microoxic conditions with the group 1b [NiFe] hydrogenase (Mohammadi *et al*. 2019), whereas *Methylacidiphilum fumariolicum* SolV can respire H_2_ at wide range of oxygen concentrations catalysed by the group 1d and group 1h [NiFe] hydrogenases (Mohammadi *et al*. 2017a). In addition, all isolates carry a gene encoding a sulfide:quinone oxidoreductase (SQR), indicating that verrucomicrobial methanotrophs might even grow in acidic ecosystems where H_2_S is present and CH_4_ and H_2_ are absent.

The main difference between *Methylacidiphilum* and *Methylacidimicrobium* strains seems to be their growth temperature, with the former being thermophiles and the being latter mesophiles. However, the isolation of *Methylacidimicrobium thermophilum* AP8 with an optimum growth temperature of 50°C muddles this clear division (Picone 2020). Still, *Methylacidiphilum* and *Methylacidimicrobium* strains are often found within a few meters of each other, but at different temperatures (Sharp *et al*. 2014). In the environment, the concentration of ammonium could be a key factor in determining which verrucomicrobial methanotrophs are present. In general, ammonia is a competitive inhibitor of pMMO, potentially reducing methane oxidation rates. Additionally, high ammonium concentrations can reduce the methane oxidation rate of microorganisms that are unable to detoxify the oxidised products of ammonia oxidation (D'Alessandro *et al*. 2009; Nyerges, Han and Stein 2010). Since only *Methylacidiphilum* strains possess a gene encoding a hydroxylamine oxidoreductase, they are well-equipped to detoxify hydroxylamine, intracellularly produced through ammonia oxidation by pMMO. Consequently, *Methylacidimicrobium* strains are expected to be primarily found at moderate temperatures and habitats with relatively low ammonium concentrations. However, different pMMOs could have different affinities for ammonia, or could not be able to oxidise ammonia at all (Nyerges and Stein 2009).

Little is known about the interaction of verrucomicrobial methanotrophs with other microorganisms in their natural geothermal habitat. Trough δ^13^C analysis of CH_4_, the origin of this gas in geothermal systems can be deduced, since CH_4_ produced by archaea is relatively light compared to thermogenic or abiotic methane (Op den Camp *et al*. 2009). Typically, methane emitted from geothermal ecosystems is from a non-microbial source, produced through a chemical reaction of H_2_ and CO or by the non-microbial decomposition of buried organic matter (Giggenbach 1995; Etiope and Klusman 2002; Fiebig *et al*. 2007). However, methanogenic archaea have been found in anoxic parts of several geothermal ecosystems, but mostly at a pH of 5 or higher (Zeikus, Ben-Bassat and Hegge 1980; Berghuis *et al*. 2019). Remarkably, a metagenome-assembled genome (MAG) closely related to the methanogen *Methanocella conradii* was abundantly present in the metagenome of the hot, acidic, and methane-rich geothermal soil on the island Pantelleria, Italy (Picone *et al*. 2020a). This finding suggests that a larger part of methane emitted from hot and acidic geothermal ecosystems could be of microbial origin than initially thought (D'Alessandro *et al*. 2009). In contrast, H_2_ utilised by verrucomicrobial methanotrophs does not seem to be produced by other microorganisms in their habitat, but rather through abiotic or thermogenic processes in the Earth's crust (Aragno 1992; Lindsay *et al*. 2019).

## CONCLUDING REMARKS AND FUTURE PERSPECTIVES

Since the isolation of verrucomicrobial methanotrophs from hot and acidic geothermal ecosystems 13 years ago, significant progress had been made in our understanding of these microorganisms living in extreme environments. Verrucomicrobial methanotrophs are much more than their name suggests: these extremophiles are actually metabolically versatile microorganisms. In fact, the same could be true for their proteobacterial counterparts. Whereas several verrucomicrobial methanotrophs were shown to grow as Knallgas bacteria, hydrogen consumption by proteobacterial methanotrophs has only been demonstrated as part of a mixotrophic lifestyle. However, proteobacterial methanotrophs that possess the CBB cycle and an uptake hydrogenase could be Knallgas bacteria as well. In recent years, scientists have mainly focused on the metabolism of inorganic compounds by verrucomicrobial methanotrophs, not of organic compounds, as was done for several proteobacterial methanotrophs. Originally thought to be obligate methanotrophs, a large variety of multi-carbon compounds such as acetate, succinate and ethanol were shown to be consumed by alphaproteobacterial methanotrophs (Dunfield and Dedysh 2014). The first attempts to grow verrucomicrobial methanotrophs on several organic compounds such as glucose, citrate and malate were unsuccessful, but later *Methylacidiphilum fumariolicum* SolV was shown to grown on ethane and butane (Op den Camp *et al*. 2009; Picone *et al*. 2020b). The major questions for future research regarding metabolism are whether proteobacterial methanotrophs can grow as Knallgas bacteria and whether verrucomicrobial methanotrophs can incorporate organic compounds into their diet.

Since multiple verrucomicrobial methanotrophs were shown to be excellent hydrogen-oxidisers, it would be worthwhile to see if new enrichment strategies using hydrogen gas, carbon dioxide and low oxygen would result in novel verrucomicrobial isolates. The exciting discovery of 16S rRNA genes related to verrucomicrobial methanotrophs outside of terrestrial volcanic ecosystems suggests a distribution outside of geothermal habitats. Several factors such as pH and temperature, but also the oxygen and ammonium concentration and the presence of methane, hydrogen gas, hydrogen sulfide and lanthanides could be used as parameters to predict the presence of verrucomicrobial methanotrophs in nature. Methane and hydrogen gas seem to be the main energy sources for verrucomicrobial methanotrophs. In addition, hydrogen sulfide could be a potent energy source, but additional physiological and biochemical experiments are needed to clarify this. From soil it is known that up to 90% of methane produced in these systems is consumed before it reaches the atmosphere, thereby contributing significantly to the mitigation of greenhouse gas emissions from the biosphere (Oremland and Culbertson 1992; Singh *et al*. 2010). To what extent verrucomicrobial methanotrophs alleviate methane emissions from geothermal environments is unknown. With the annual seepage of 40 to 60 Tg methane from mud volcanoes, fumaroles and hydrothermal vents (Etiope 2009), it would be interesting to investigate verrucomicrobial methanotrophy in the environment, especially if the use alternative energy sources such as hydrogen gas and hydrogen sulfide could enhance this process.

## Supplementary Material

fuab007_Supplemental_File
